# Design and Analysis of Wideband Single-Layer Reflectarray Antenna for Remote Sensing and Environmental Monitoring

**DOI:** 10.3390/s25030954

**Published:** 2025-02-05

**Authors:** Annal Joy J, Sandeep Kumar Palaniswamy, Sachin Kumar, Malathi Kanagasabai, Ladislau Matekovits

**Affiliations:** 1 Department of Electronics and Communication Engineering, Faculty of Engineering and Technology, SRM Institute of Science and Technology, Kattankulathur 603203, Tamil Nadu, India; jannaljoy@gmail.com; 2 Department of Electronics and Communication Engineering, Galgotias College of Engineering and Technology, Greater Noida 201310, Uttar Pradesh, India; gupta.sachin0708@gmail.com; 3 Department of Electronics and Communication Engineering, College of Engineering, Guindy, Anna University, Chennai 603103, Tamil Nadu, India; mala@annauniv.edu; 4 Department of Electronics and Telecommunications, Politecnico di Torino, 10129 Turin, Italy; 5 Faculty of Electronics and Telecommunications, Politehnica University Timişoara, 300223 Timişoara, Romania; 6 Istituto di Elettronica e di Ingegneria dell’Informazione e delle Telecomunicazioni, National Research Council of Italy, 10129 Turin, Italy

**Keywords:** high gain, phase range, reflectarray antenna, satellite communication, wideband

## Abstract

In this article, a wideband single-layer reflectarray antenna for Ku-band applications is presented. The proposed reflectarray antenna is suitable for applications such as fixed satellite service (FSS), broadcasting satellite service (BSS), earth exploration satellite service (EESS), remote sensing, and environmental monitoring. The developed single element of the proposed reflectarray antenna is made up of a horizontal strip, discrete vertical strips of varying sizes, and circular structures. The reflectarray antenna has 441 elements arranged on a square aperture made of Rogers 5880 substrate, measuring 21 cm × 21 cm. The maximum gain obtained is 26.31 dBi, with a bandwidth of 15.4% of 1 dB gain. The achieved aperture efficiency is 44.4%. The obtained cross-polarizations are less than −21.46 dB for the E-plane and −25.27 dB for the H-plane. The side lobe level is found below −15.06 dB in the E plane and −15.7 dB in the H plane. The side lobe level is minimal at 13.5 GHz, measuring less than −18.2 dB and −18.5 dB in the E and H planes, respectively. The reflectarray antenna designed has a fractional bandwidth of 40%. Hence, the developed antenna is suitable for wide Ku-band applications.

## 1. Introduction

Satellites have become indispensable for day-to-day activities such as communication, navigation, and weather forecasting. Fixed satellite service (FSS), earth exploration satellite service (EESS), and broadcasting satellite service (BSS) play major roles in daily lives by providing endless communication between two fixed points on earth. The earth exploration satellite service collects data on the earth’s environmental and atmospheric conditions, while the broadcast satellite service provides entertainment and television services to the general public [1]. There are many environmental changes these days, and therefore remote sensing is required for monitoring environmental conditions. The collected information is then transmitted to satellite communication systems for processing, analysis, and interpretation. Without satellite communication, remote sensing data from space would be difficult to transmit in real time or over long distances.

High-gain Ku-band antennas are required for such communication systems, as they are excellent candidates for long-distance transmission [2]. Reflector and array are examples of conventional high-gain antennas, but they have disadvantages such as a high geometric profile, difficulty achieving a flexible beam, and complex feeding networks [3]. Reflectarray antennas can overcome these limitations because they have a simple profile and low fabrication costs and are easy to deploy [4]. Reflectarray antennas are array antennas with a spatial feed [5]. The reflectarray antenna, combining the advantages of both reflector and array antennas, is a viable alternative to conventional parabolic reflectors [6,7]. Reflectarray antennas are important components in remote sensing and satellite communication, as they enable efficient signal transmission and beam steering.

Researchers have focused on developing single-layer, multi-layer, single-feed, multi-feed, single-band, multi-band, and wideband high-gain reflectarray antennas to meet their needs for long distance transmission, such as satellite communication. A multi-layer Minkowski fractal reflectarray is developed in [8] to address phase error. Even though the required phase range is obtained, the antenna profile is bulky. In [9], a multi-layer reflectarray antenna is designed to achieve high gain, better performance, and wideband characteristics. Although multi-layer is used for high performance, the side lobe level is less than −10.4 dB. In [10], a multi-layer dual-band reflectarray antenna with a frequency selective surface (FSS) is used to operate at the X and Ka bands, but this increases the antenna’s complexity. In [11], a multi-feed reflectarray antenna is developed, and a phasing technique is used to achieve wide beam angle performance at the cost of complexity. For a reflectarray antenna, the unit cell must produce a phase range of 360° or more. If the phase range of the reflectarray antenna’s unit cell is less than 360°, phase error occurs, and an efficient reflectarray antenna cannot be built. In [12], a phase range of 360° is obtained by varying the gap of the element. Changing the length of the cross-dipole in [13], a phase range of 360° at 30.2 GHz and 350° at 20.4 GHz is obtained. The required phase range is not achieved at 20.4 GHz, and therefore the developed antenna is not suitable for 20.4 GHz applications.

One significant disadvantage of reflectarray antennas is their narrow bandwidth. Therefore, it is critical to consider the antenna’s bandwidth [14]. Many researchers have focused on developing single- and multi-band reflectarray antennas. In [15], the proposed reflectarray antenna operates in three different bands (C, X, and Ku), but its bandwidth is limited. The antenna operates at 3.9, 7.5, and 12.5 GHz, resulting in narrow bands. In [10], the developed antenna operates at 20 GHz (19.6–21.2 GHz) and 30 GHz (29.4–31 GHz). Even though the antenna supports dual-band operation, the bandwidth covered is limited. Another important parameter of a reflectarray antenna is its gain. If the gain of the antenna is low, it will be unable to cover long-distance communication. In [16], an angular ring-shaped reflectarray antenna element is developed, yielding a peak gain of 19.4 dBi. A peak gain of 21.5 dBi is achieved at 32 GHz with a single-layer folded reflectarray antenna [17]. In order to design a high-performance antenna, the side lobes must be kept to a minimum. In [18], the measured side lobe levels are less than −13 dB. The side lobe levels are found to be higher (−10 dB) in [3,12,19], which affects the antenna’s performance.

The literature shows that using a multi-layer, multi-feed reflectarray increases the antenna’s complexity and the performance is still not optimal. Choosing the appropriate phasing technique and unit cell structure remains difficult, as some techniques and structures fail to produce the required phase range. Many researchers have worked on developing a multi-band reflectarray antenna but have not achieved wideband performance. As reported in the literature, it is difficult to select an appropriate number of elements and achieve a high gain. Some research suggests that reducing unwanted side lobe levels is difficult. Taking all of this into account, this article describes the development of a single-layer wideband reflectarray antenna for Ku-band applications. The proposed antenna operates from 12 to 18 GHz and supports FSS, BSS, EESS, remote sensing, and environmental monitoring. A phase range of 498° is achieved by selecting the optimized unit cell structure and varying the size of the vertical strips. For a wideband antenna, the required fractional bandwidth is more than 20%. The proposed reflectarray antenna has a 40% fractional bandwidth, making it a wideband antenna. The peak gain is 26.3 dBi, and the antenna is able to communicate over long distances. The obtained side lobe and cross-polarization levels are satisfactory for an efficient reflectarray antenna. Furthermore, free space path loss (FSPL) and link budget are computed to understand the loss in signal strength and coverage.

## 2. Design and Analysis of the Single Element

**Figure 1:**
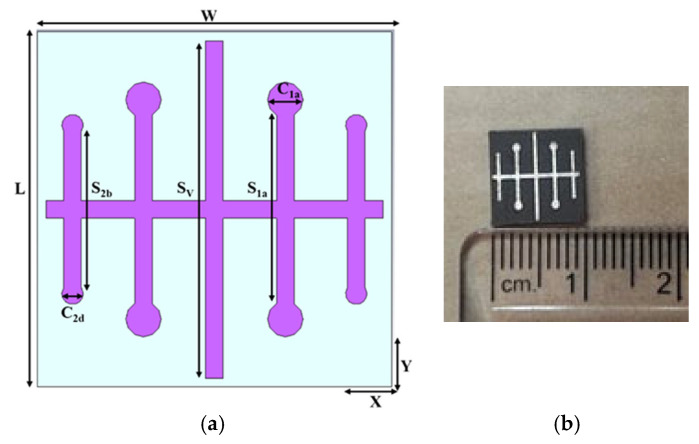
Unit cell: (**a**) structure; (**b**) photograph of the fabricated prototype.

Figure 1 depicts the proposed single element and the fabricated prototype. The developed unit cell is made up of five distinct vertical strips of varying lengths and a horizontal strip. The vertical middle strip (denoted as *S_V_*) is longer than the four other vertical strips. The vertical strips adjacent to the middle strip on both sides are 3.325 mm smaller than the middle strip and are referred to as *S*_1*a*_ and *S*_1*b*_. One more strip is placed next to *S*_1*a*_, and the other strip is placed next to *S*_1*b*_, and these are referred to as *S*_2*a*_ and *S*_2*b*_. *S*_2*a*_ and *S*_2*b*_ are 4.75 mm shorter than the middle strip. *S*_1*a*_ and *S*_1*b*_ are attached to two circles on either side of the strips. The quad circles have a diameter of 1 mm and are designated as *C*_1*a*_, *C*_1*b*_, *C*_1*c*_, and *C*_1*d*_. The *S*_2*a*_ and *S*_2*b*_ are attached to 0.6 mm circles on either side of their strip, which are known as *C*_2*a*_, *C*_2*b*_, *C*_2*c*_, and *C*_2*d*_. The distance between the strips is 1.5 mm. A horizontal strip, the same size as the vertical middle strip, is placed in the center.

Table 1 highlights the dimensions of the constructed unit cell. The unit cell structure is printed on a Rogers 5880 substrate with a dielectric constant of 2.2, loss tangent of 0.0009, and a thickness of 1.52 mm. The element has dimensions of 10 mm × 10 mm. CST Microwave Studio Suite v. 2024 software is used for the electromagnetic simulations. The developed unit cell operates in the 12–18 GHz Ku-band. The proposed antenna covers remote sensing in the Ku-band, satellite communication (uplink) from 14 to 14.5 GHz, FSS downlink from 12.5 to 12.75 GHz and FSS uplink from 14 to 14.5 GHz, BSS uplink from 17.3 to 17.8 GHz, EESS from 13.4 to 13.75 GHz, radar application from 13.4 to 14 GHz, and maritime satellite communication (uplink) from 14 to 14.5 GHz.

### 2.1. Evolution of the Single Element

**Figure 2:**
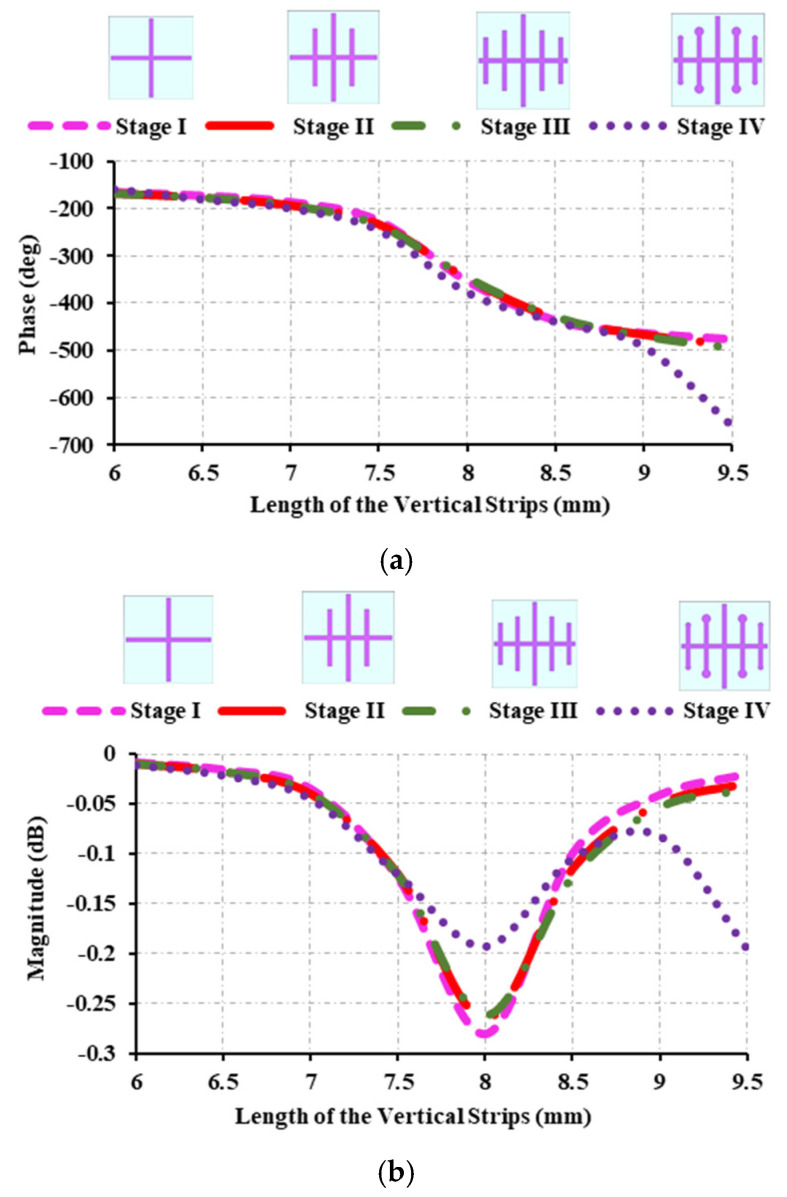
Evolution stages of the developed reflectarray antenna: (**a**) reflection phase; (**b**) reflection magnitude.

Figure 2 depicts the evolutionary stages of the proposed single element and its corresponding reflection characteristics. The unit cell phase range is an important parameter to consider when constructing a reflectarray antenna. If the unit cell generates the desired phase range, the single element can be converted into a reflectarray antenna. For a reflectarray antenna, the required phase range should be equal to or more than 360°. If the phase range is below 360°, phase error occurs. It is necessary to develop a reflectarray antenna’s unit cell without phase error. Figure 2a depicts the design stage I of the developed element. The design began with a symmetrical cross structure. The variable size method is employed to achieve the desired phase range. The length of the vertical strip of the cross structure is varied, and a phase range of 311° is achieved.

Since the desired phase range is not achieved, two vertical strips on either side of the middle strip are added in design stage II, and the lengths of the three strips are adjusted, resulting in a 325° phase range. To increase the phase range, two more vertical strips of length 4.75 mm are introduced, resulting in a phase range of 335°. To avoid phase error and broaden the phase range, two circles of varying sizes are added on either side of the strips, with the exception of the middle strip, achieving a phase range of 498°. Particularly, in stage IV, the phase error has been eliminated and a wide range of phase has been achieved. As the obtained phase range is wide, the response of the phase is smooth and there will be fewer abrupt changes in the design, which results in an efficient reflectarray antenna. Figure 2b depicts the reflection magnitude obtained at each evolutionary stage. The reflection magnitude is greater than 0 dB but less than 0.27 dB at all stages.

### 2.2. Parametric Study of the Unit Cell

**Figure 3:**
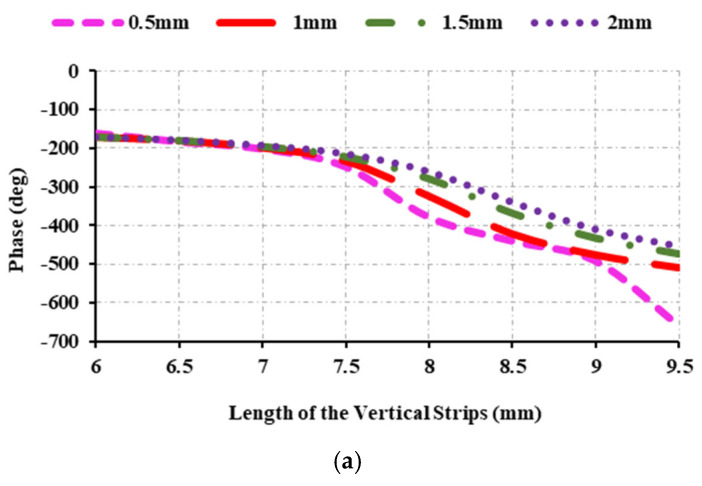

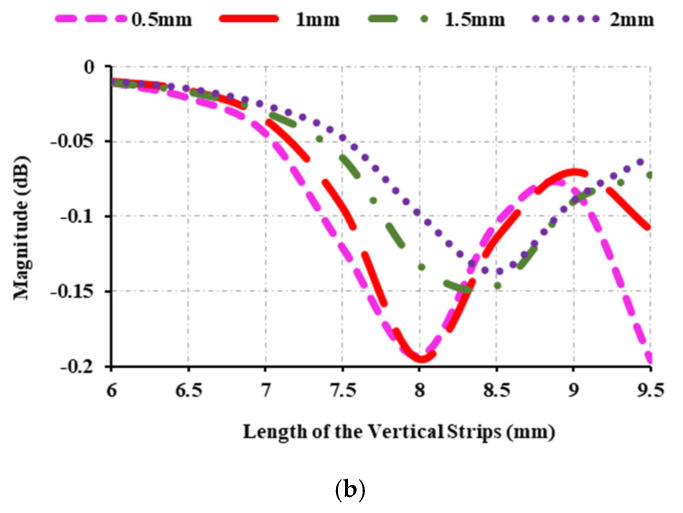
Reflection characteristics at 12.5 GHz: (**a**) variations of reflection phase and width; (**b**) variations of reflection magnitude and width.

To design an efficient unit cell, the parameters of the unit cell are optimized. Therefore, different parameters of the unit cell are varied, and its reflection characteristics are investigated in order to determine the optimal value for the width of the horizontal strip, diameter of the circles on the first and second strips, spacing between the vertical strips, and angle of incidence of the proposed unit cell. First, the width of the horizontal strip is varied to achieve the optimal width. The width of the horizontal strip is changed from 0.5 mm to 2 mm, and the corresponding reflection characteristics are calculated and plotted in Figure 3. When the strip width is 2 mm, the phase range is 282°; at 1.5 mm, the phase range is 302°; when the strip width is 1 mm, the phase range is 339°; and at 0.5 mm, the phase range is 498°. This demonstrates that when the width of the strips is 1 mm, 1.5 mm, or 2 mm, the required phase range is not achieved. The required phase range is seen at 0.5 mm. Thus, the optimal width of the strip is 0.5 mm. It should also be noted that the phase range decreases as the width of the strips increases. Figure 3b shows the magnitude of the element at various strip widths, and it can be seen that it is greater than 0 dB but less than −0.2 dB.

**Figure 4:**
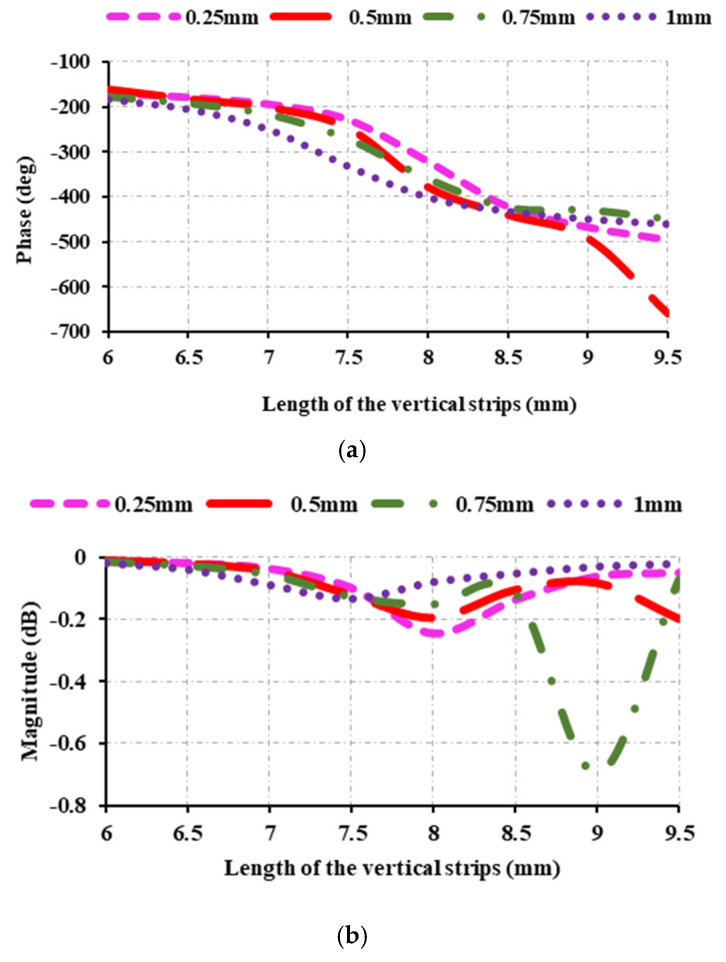
Reflection characteristics at 12.5 GHz: (**a**) variations of reflection phase and circle diameter (*C_a_*_,*b*_) on the first right and left strips; (**b**) variations of reflection magnitude and circle diameter (*C_a_*_,*b*_) on the first right and left strips.

In Figure 4, the reflection characteristics are investigated by varying the diameter of the circle on the first right and left vertical strips. The diameter of the circles on the first strips is varied between 0.25 mm, 0.5 mm, 0.75 mm, and 1 mm, and it is found that 0.5 mm is the optimal diameter. A phase range of 327° is obtained when the circle, *C_a_*_,*b*_, has a diameter of 0.25 mm. The phase range at 0.75 mm is 273°, and at 1 mm it is 277°. A phase range of 498° is achievable with a diameter of 0.5 mm. This shows that the phase range decreases above 0.5 mm and below 0.5 mm.

**Figure 5:**
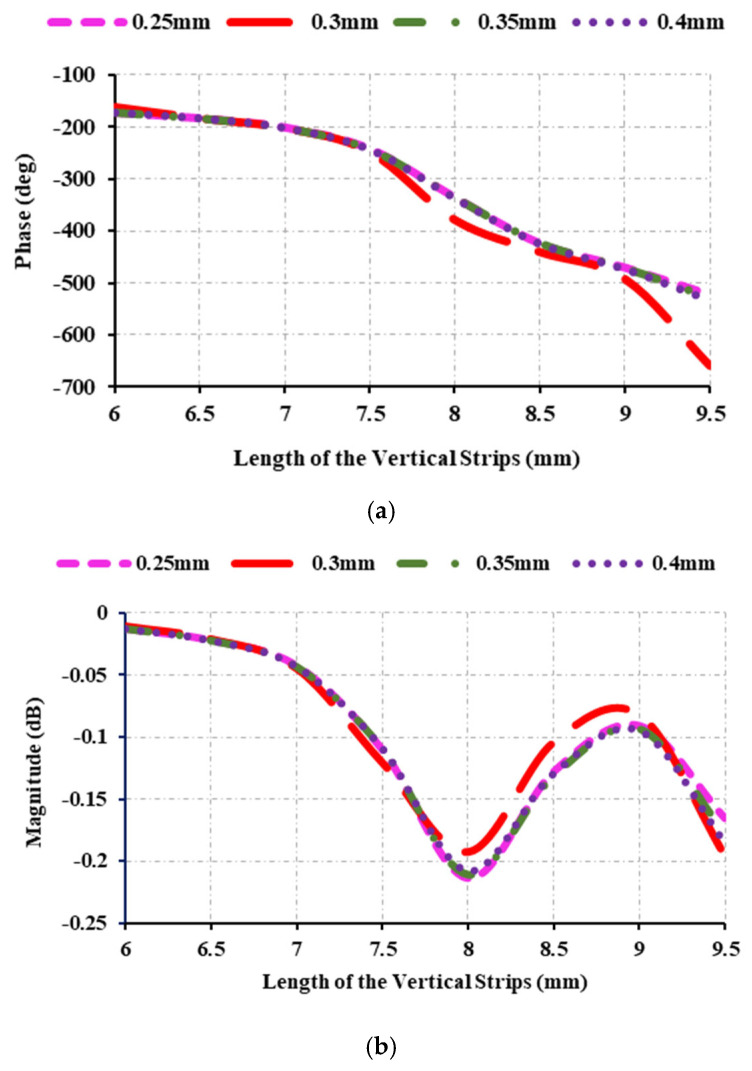
Reflection characteristics at 12.5 GHz: (**a**) variations of reflection phase and circle diameter (*C_c_*_,*d*_) on the second right and left strips; (**b**) variations of reflection magnitude and circle diameter (*C_c_*_,*d*_) on the second right and left strips.

Figure 4b shows that the magnitude of the unit cell drops up to −0.7 dB at 0.75 mm. However, the magnitude of the unit cell is kept between 0 dB and −1 dB, ensuring that the element maintains its required magnitude. The diameter of the circles on the second vertical strip (*C_c_*_,*d*_) is also adjusted, and the reflection properties are examined. The diameter of the circle varies from 0.25 mm to 0.4 mm. Figure 5 shows that the phase range from 0.25 mm to 0.4 mm is relatively uniform. The phase range varies slightly across dimensions, with the maximum phase range occurring at 0.3 mm. Similarly, the magnitude ranges between 0 dB and −0.25 dB in all cases.

**Figure 6:**
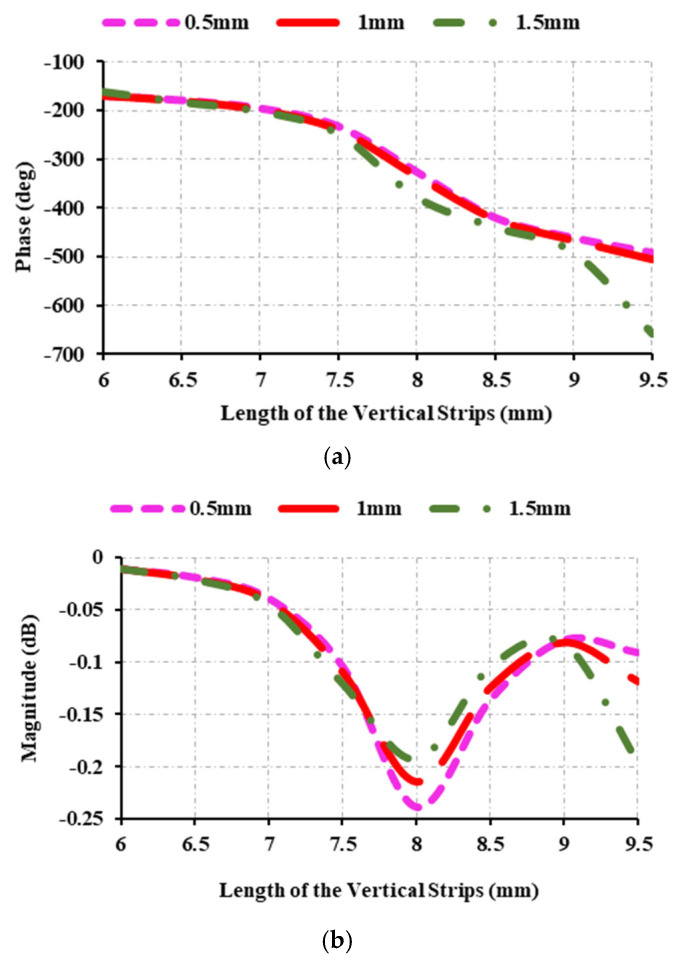
Reflection characteristics at 12.5 GHz: (**a**) variations of reflection phase and spacing between vertical strips; (**b**) variations of reflection magnitude and spacing between vertical strips.

In Figure 6, the reflection parameters of the unit cell are investigated by varying the spacing between the vertical rods. The reflection parameter is examined when the spacing between the elements is 0.5 mm, 1 mm, or 1.5 mm. The achieved phase is found to be 324°, 335°, and 498° at 0.5 mm, 1 mm, and 1.5 mm, respectively. Consequently, the vertical strip spacing remains constant at 1.5 mm. In Figure 6b, the magnitude in all cases ranges from 0 dB to 0.25 dB. Changing the angle of incidence allows one to understand the reflection features of the unit cell.

**Figure 7:**
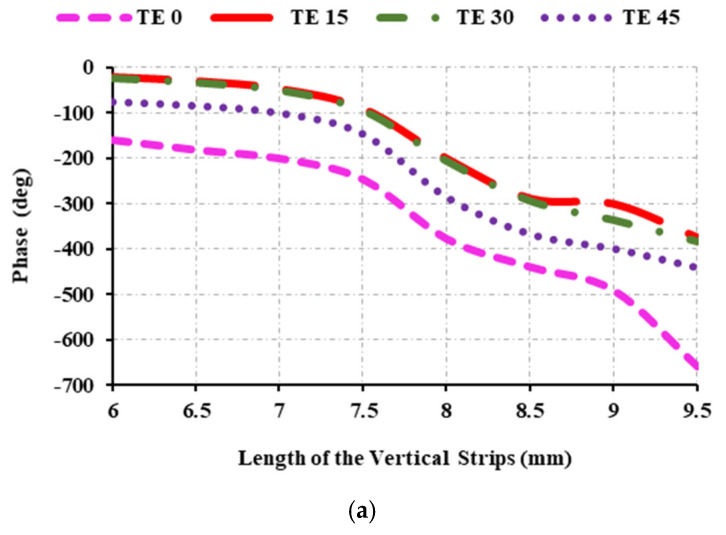

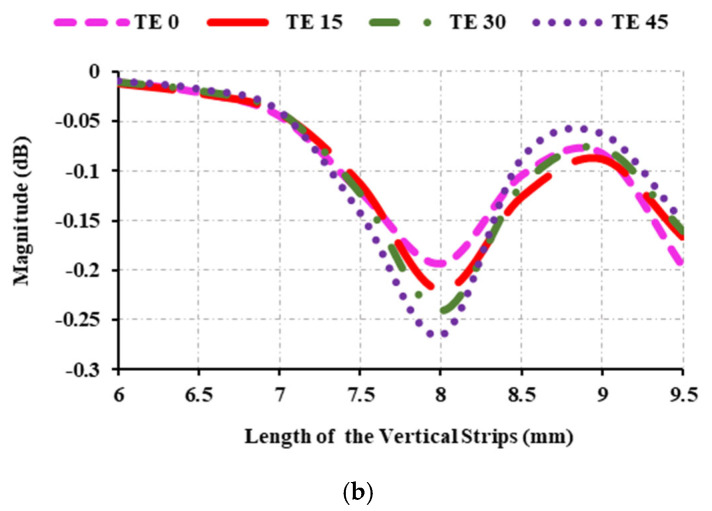
Reflection characteristics at 12.5 GHz: (**a**) variations of reflection phase and angle of incidence; (**b**) variations of reflection magnitude and angle of incidence.

Figure 7 shows the reflection curve when *ɸ* is set to 0 and *θ* is varied among 0, 15, 30, and 45. At TE 0, the unit cell produces a better reflection phase, with the magnitude ranging from 0 dB to −0.3 dB. Table 1 displays the optimal parametric values derived from the investigation.

### 2.3. Reflection Characteristics of the Proposed Element

**Figure 8:**
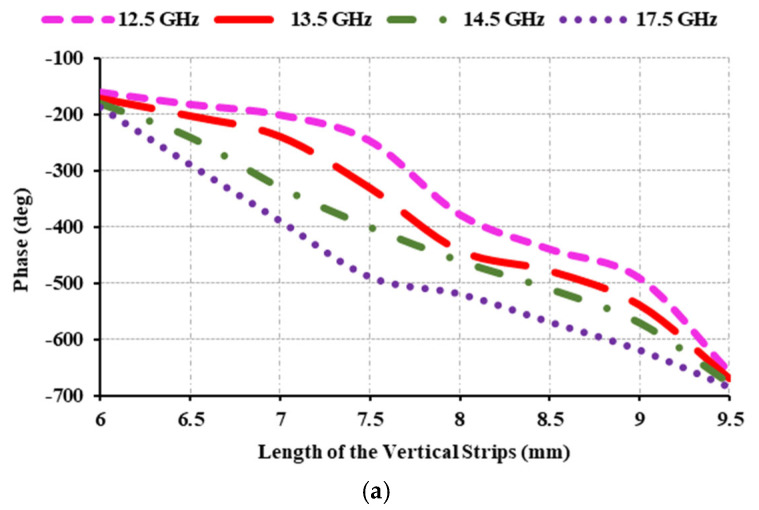

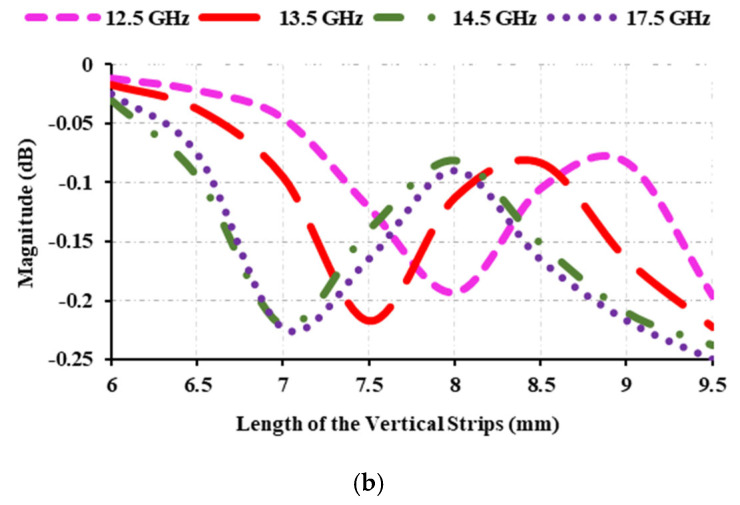
Reflection characteristics of the element: (**a**) phase (degree); (**b**) magnitude (dB).

The unit cell is developed using the parametric values listed in Table 1. Figure 8 depicts the phase range and magnitude of the proposed element. Figure 8 shows the obtained reflection characteristics at 12.5 GHz, 13.5 GHz, 14.5 GHz, and 17.5 GHz. Since the phase curves for the specified frequencies are parallel, the developed unit cell produces wideband.

Figure 8b depicts the magnitude of the element at various frequencies, and it is found that the magnitude achieved for all of the mentioned frequencies is less than 0 and greater than −0.25 dB. It is evident that the obtained phase range is 498°. Thus, the proposed unit cell meets the basic requirements for conversion into a reflectarray antenna.

### 2.4. Surface Current

**Figure 9:**
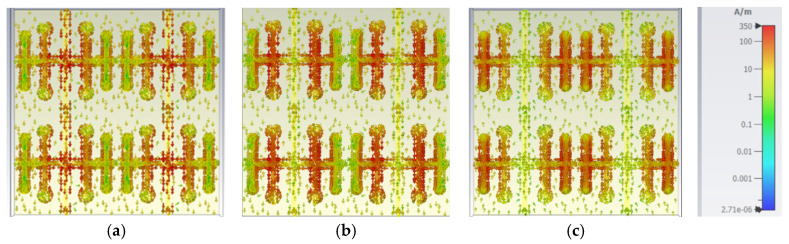
Surface current distribution of the element: (**a**) 12 GHz; (**b**) 15 GHz; (**c**) 18 GHz.

The surface current of the developed element is displayed in Figure 9a–c. Figure 9a depicts the surface current at 12 GHz, demonstrating that the current density is highest in the center of the vertical strip. In Figure 9b, at 15 GHz, the current density is found to be greater in both the first vertical strip that is located adjacent to the left side and right side of the middle strip. Figure 9c shows that the current density is higher in the second vertical strip on both the right and left side at 18 GHz. Thus, the entire element aids in achieving resonance at various frequencies.

## 3. Construction of the Reflectarray Antenna

**Figure 10:**
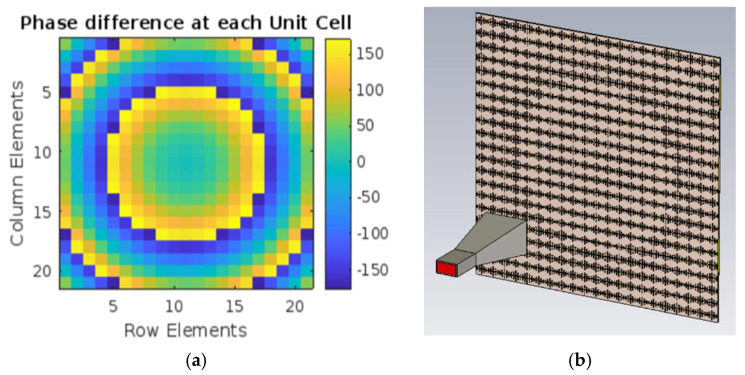
Reflectarray antenna: (**a**) phase compensation for 21 × 21 reflectarray antenna; (**b**) designed 21 × 21 reflectarray antenna.

Since the unit cell has the required phase range, it is converted into a reflectarray antenna for Ku-band applications. The reflectarray antenna is constructed on the Rogers RT Duroid 5880 substrate with a dielectric constant of 2.2, a loss tangent of 0.0009, and a thickness of 1.52 mm. The reflectarray antenna has 441 elements in a 21 × 21 array, with a square aperture of 210 mm × 210 mm. The wideband reflectarray antenna is fed spatially by the feed horn. Equation (1) is used to compute the required phase compensation for each element.

(1) $${ɸ}_{mn}=k_{0}(d_{i}-(x_{i}cos\varphi_{0}+y_{i}sin\varphi_{0})\times sin\;\theta_{0})$$

where $k_{0}$ is the propagation constant, and $d_{i}$ represents the distance between the phase center of the feed horn and the center of the *i*th element on the developed wideband reflectarray antenna. The main beam direction is $\left(\theta_{0},\;\varphi_{0}\right)$. The required phase compensation is calculated with MATLAB version 2024a, and the phase compensation matrix is shown in Figure 10a. The size and location of 21 × 21 elements are determined by comparing phase compensation values to the phase curve. The 441 elements are arranged on the Rogers RT Duroid 5880 substrate, with a ground plane behind it. The elements are spaced apart by 10 mm.

Figure 10b depicts the designed reflectarray antenna. The reflectarray is fed by a horn antenna with a focal distance of 21 cm. The *f*/*D* ratio determines the distance between the reflector array and the feed horn. Here, *f* denotes the focal distance and *D* denotes the diameter of the reflectarray aperture. The focal distance between the horn and the reflectarray antennas has a significant impact on their gain and frequency. The focal distance between the horn and the reflectarray is varied to 12.6 cm, 16.8 cm, and 21 cm to achieve the best position for the feed horn. These distances are based on *f*/*D* ratios of 0.6, 0.8, and 1.

**Figure 11:**
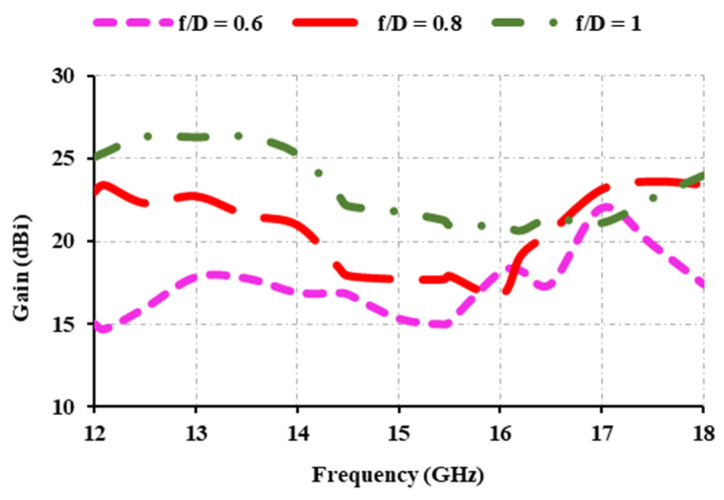
Gain of the proposed antenna at various *f*/*D* ratios.

The effect of the *f*/*D* ratio is displayed in Figure 11. It can be seen that when the *f*/*D* ratio is 0.6, the gain is greater than 15 dBi; when the *f*/*D* ratio is 0.8, the gain is greater than 16 dBi; and when the *f*/*D* ratio is 1, the gain exceeds 20 dBi. Thus, a focal distance of 21 cm is maintained between the reflectarray and the horn antenna.

### 3.1. Radiation Characterization

**Figure 12:**
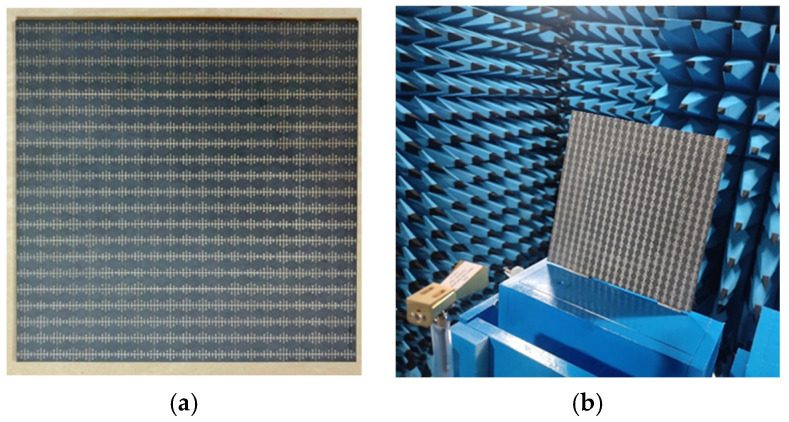
Fabricated prototype: (**a**) 21 × 21 reflectarray; (**b**) measurements in an anechoic chamber.

This section discusses the antenna characteristics obtained by simulating the antenna in a time domain with open boundary conditions. Figure 12a depicts the prototype of the proposed antenna. Figure 12b depicts the measurement set-up for the proposed reflectarray antenna in an anechoic chamber. The Ku-band performance of the antenna is measured by positioning the pyramidal receiving horn 21 cm away from the prototype. The antenna works from 12 to 40 GHz, with a 15.3 dB gain at 15 GHz and a beam width of 22°. The radiation pattern is measured by placing the proposed antenna in an anechoic chamber and computing the pattern using a computer.

**Figure 13:**
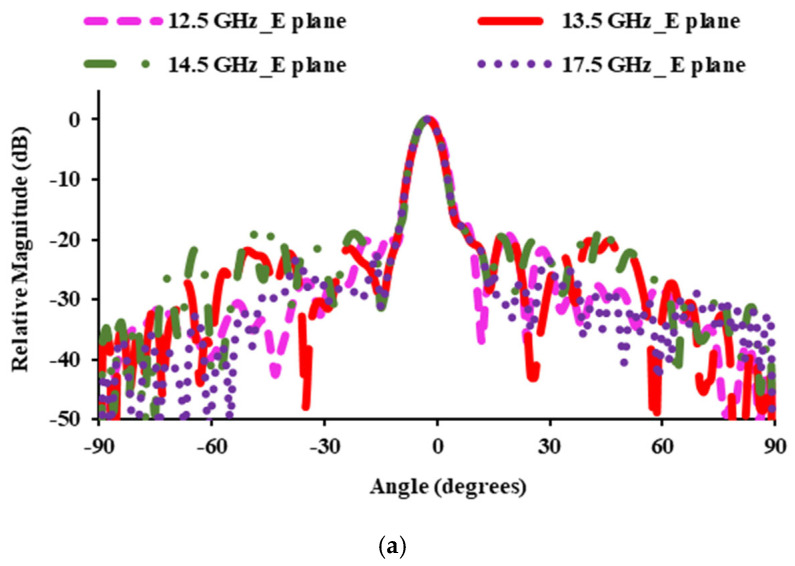

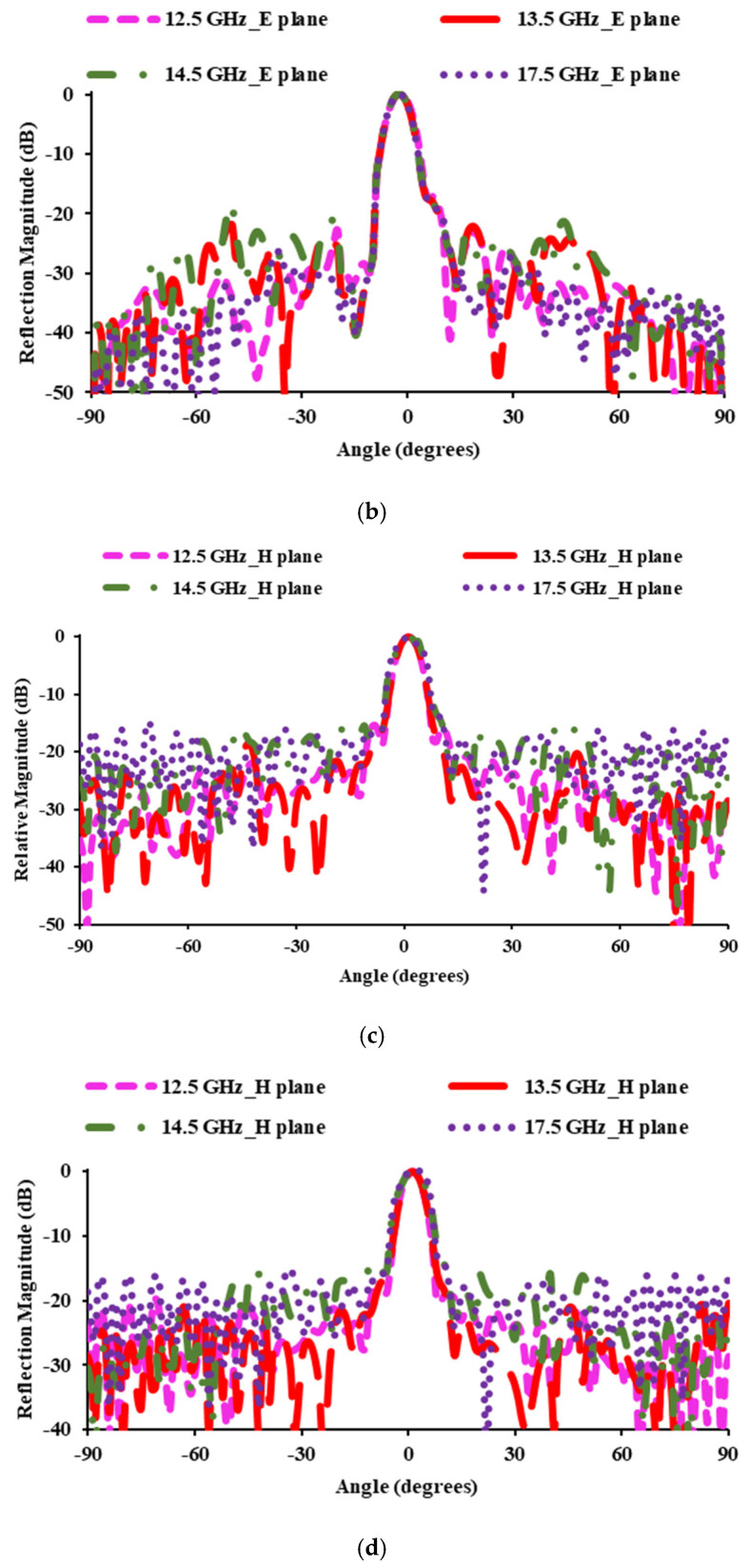
Radiation patterns: (**a**) simulated co-polarization in the E plane; (**b**) simulated co-polarization in the H plane; (**c**) measured co-polarization in the E plane; (**d**) measured co-polarization in the H plane.

Figure 13 shows the simulated and measured (E plane and H plane) radiation patterns at 12.5 GHz, 13.5 GHz, 14.5 GHz, and 17.5 GHz. At 12.5 and 13.5 GHz, the relative magnitude is 26.31 dB, while at 14.5 and 17.5 GHz, it is 22.14 dB and 22.61 dB, respectively. In the E plane, the obtained side lobe level is below −17.2 dB, −18.2 dB, −17.61 dB, and −15.7 dB at 12.5 GHz, 13.5 GHz, 14.5 GHz, and 17.5 GHz, respectively. In the H plane, side lobe levels are −15.72 dB at 12.5 GHz, −18.5 dB at 13.5 GHz, −15.06 dB at 14.5 GHz, and −16.18 dB at 17.5 GHz.

**Figure 14:**
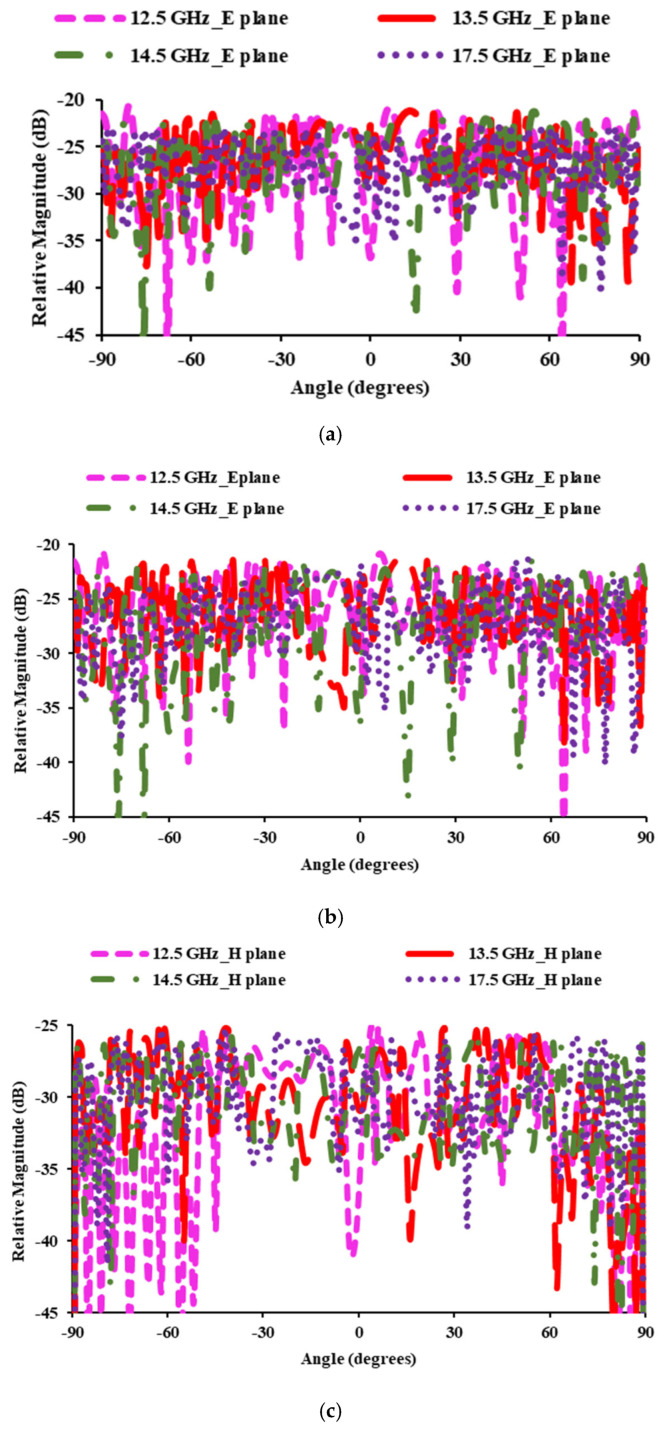

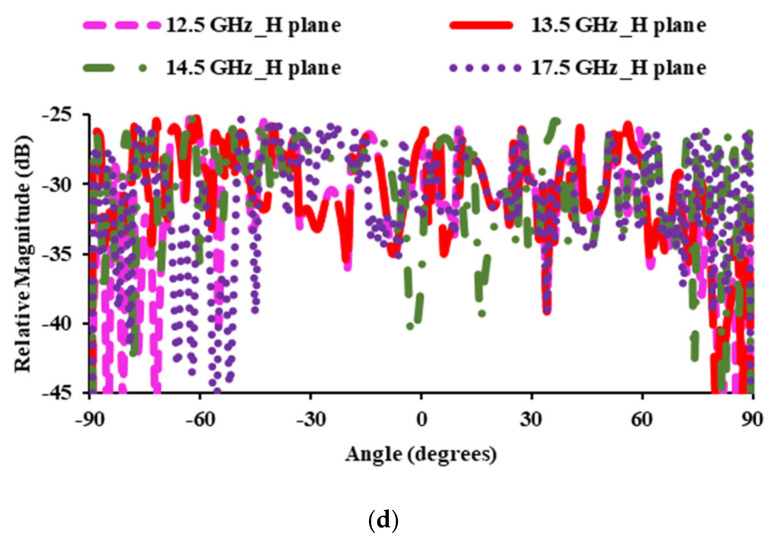
Radiation patterns: (**a**) simulated cross-polarization in the E plane; (**b**) simulated cross-polarization in the H plane; (**c**) measured cross-polarization in the E plane; (**d**) measured cross-polarization in the H plane.

Figure 14 shows the cross-polarization levels for both the E and H planes. In the E plane, cross polarization levels are less than −21.46 dB at 12.5 GHz (FSS downlink), −22 dB at 13.5 GHz (EESS), −22.03 dB at 14.5 GHz (FSS uplink), and −23.03 dB at 17.5 GHz. Cross-polarization levels of less than −25.27 dB, −25.51 dB, −26.03 dB, and −25.50 dB are achieved at 12.5 GHz, 13.5 GHz, 14.5 GHz, and 17.5 GHz, respectively.

**Figure 15:**
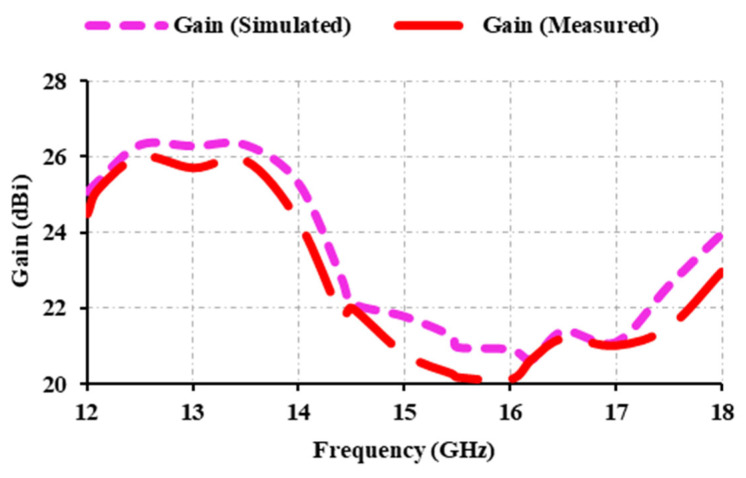
Simulated and measured gain of the proposed antenna.

**Figure 16:**
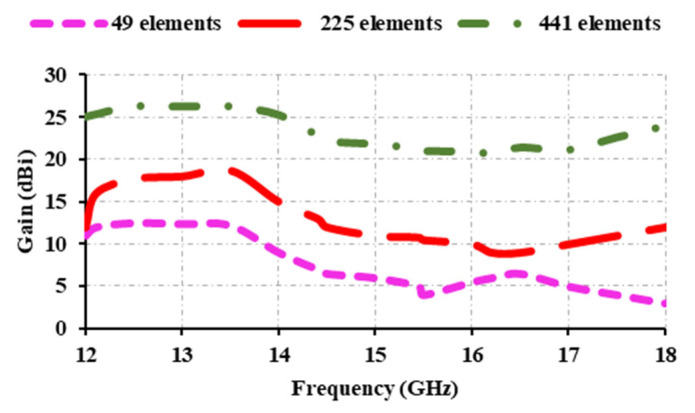
Gain of the antenna at various numbers of elements.

Figure 15 depicts the simulated and measured results of the proposed reflectarray antenna. The FSS downlink (12.5 GHz) and EESS (13.5 GHz) offer a gain of 26.31 dBi. At 14.5 GHz, the gain is 22.14 dBi, while at 17.5 GHz, it is 22.61. The peak gain is 26.31 dBi at 12.5 and 13.5 GHz. There is a slight variation in the measured gain when compared with the simulated. This is due to fabrication losses and measurement misalignment errors. The obtained 1 dB gain bandwidth is 15.4%, spanning the 12–14 GHz range. The gain from 12 GHz to 18 GHz exceeds 20.6 dBi. For any long-distance application, the antenna gain should exceed 20 dBi. In this case, the proposed antenna provides more than 20 dBi from 12 GHz to 18 GHz, spanning the entire Ku-band. Thus, the proposed antenna is suitable for long-distance applications. The number of elements used affects the gain characteristics of the antenna. The higher the number of elements used, the greater the gain, but the aperture size also increases. Thus, a trade-off exists between the number of elements used and the antenna’s gain. In Figure 16, 7 × 7, 15 × 15, and 21 × 21 elements have been compared, and it can been seen that when 49 elements are used, the peak gain obtained is 12.5 dBi; at 225 elements, maximum gain is 18.7 dBi; and at 441 elements, a peak gain of 26.3 dBi is attained. High-gain antennas are those that have a gain of more than 20 dBi. From this, it is evident that using 441 elements provides high gain compared to a lower number of elements. Taking aperture size into account, as sufficient gain has been attained, 441 elements have been chosen to construct the reflectarray antenna. The aperture efficiency of the reflectarray antenna with respect to gain in dB is given as

(2) $$A_{e}=\frac{c^{2}\times 10^{G\left(dB\right)/10}}{f^{2}\times 4\pi \times A_{p}}\times 100\%$$

Here, the physical aperture of the reflectarray antenna is denoted as $A_{p}$, and *f* is the operating frequency. The measured aperture efficiency is found to be 44.4% at 12.5 GHz.

### 3.2. Fractional Bandwidth

Fractional bandwidth is computed to understand the antenna’s bandwidth. It is defined as the difference between the higher and lower cut-off frequencies divided by the center frequency. It can be expressed using Equation (3),

(3) $$FBW=\frac{f_{2}-f_{1}}{f_{c}}$$

where *f*_2_ is the higher cut-off frequency, *f*_1_ is the lower cut-off frequency, and *f_c_* is the center frequency. Fractional bandwidth should be between 0 and 2, and is typically expressed as a percentage ranging from 0% to 200%. A wideband antenna is one that covers more than 20% of the spectrum. The proposed antenna has a higher cut-off frequency of 18 GHz, a lower cut-off frequency of 12 GHz, and a center frequency of 15 GHz, resulting in a fractional bandwidth of 40%.

### 3.3. Free-Space Path Loss (FSPL)

FSPL was investigated to determine the loss in signal strength that occurs between transmitting and receiving antennas when traveling in free space. FSPL can be expressed using Equation (4),

(4) $$FSPL=20{log}_{10}\left(d\right)+20{log}_{10}\left(f\right)+20{log}_{10}\left(\frac{4\pi }{c}\right)-G_{Tx}-G_{Rx}$$

where *d* is the distance between the transmitting and receiving antennas, *f* is the frequency, *c* is the speed of light, $G_{Tx}$ is the gain of the transmitting antenna, and $G_{Rx}$ is the gain of the receiving antenna.

**Figure 17:**
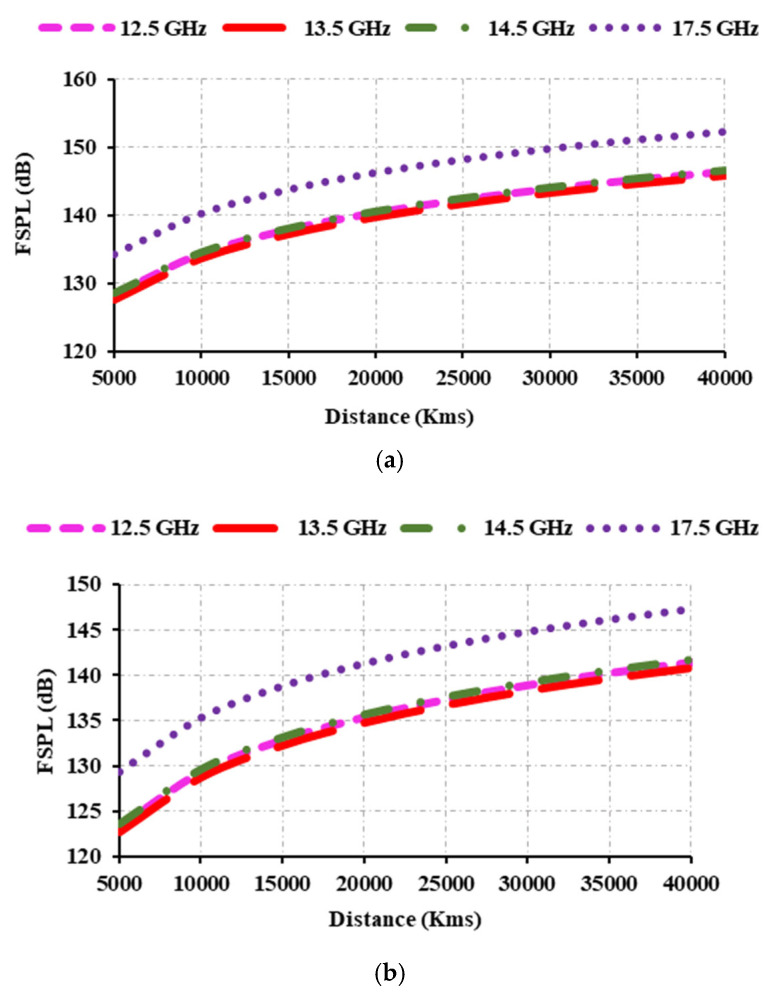

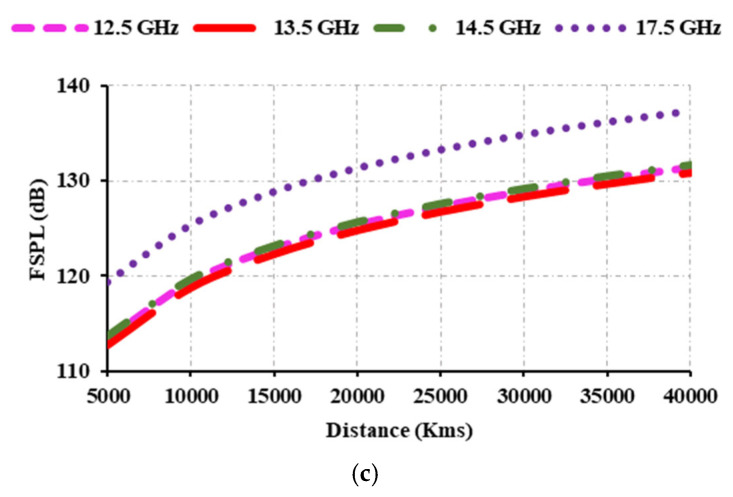
FSPL analysis by varying distance from 5000 to 40,000 km: (**a**) *R_X_* gain at 35 dBi; (**b**) *R_X_* gain at 40 dBi; (**c**) *R_X_* gain at 50 dBi.

**Figure 18:**
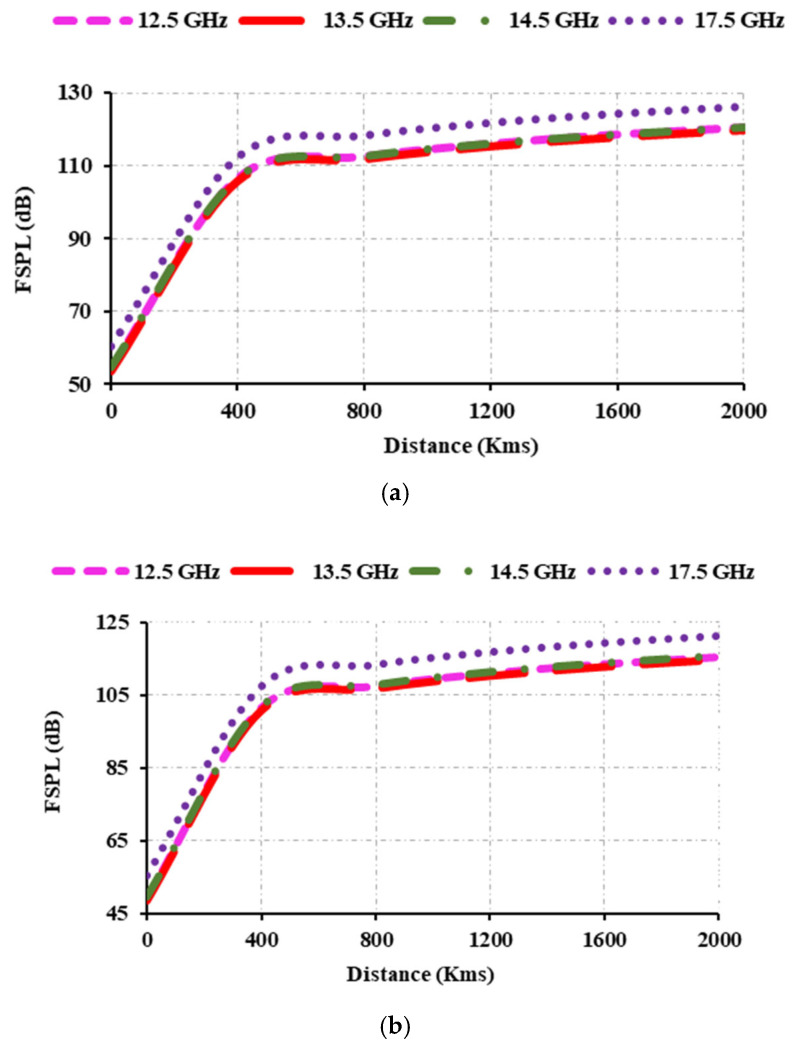

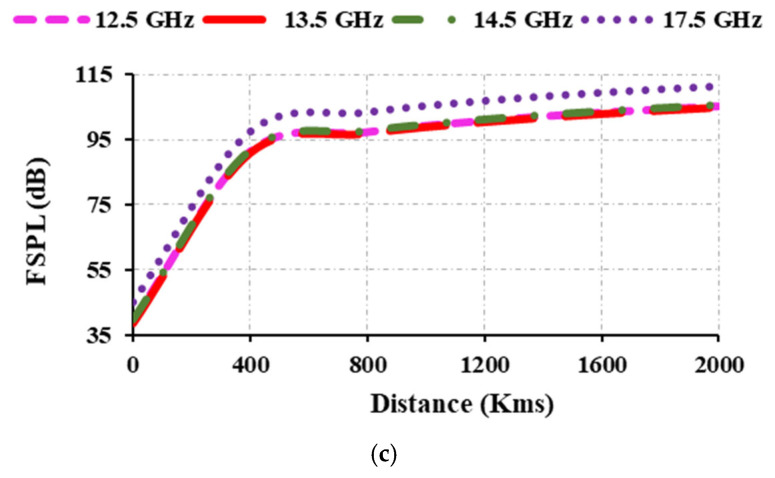
FSPL analysis by varying distance from 1 to 2000 km: (**a**) *R_X_* gain at 35 dBi; (**b**) *R_X_* gain at 40 dBi; (**c**) *R_X_* gain at 50 dBi.

When calculating FSPL, the distance between the two antennas as well as their frequency and gain are all important considerations. The distance between the transmitter and receiver antennas must be known for satellite applications such as FSS, BSS, and EESS. Typically, the antennas used in FSS, BSS, EESS, and remote sensing are placed in geostationary orbits (GEO), medium earth orbits (MEO), and low earth orbits (LEO). The antennas in GEO, MEO, and LEO serve as transmitting antennas, while the antenna in the ground station serves as a receiver antenna. The distance between the GEO and the earth station is approximately 35,786 km, the distance between the MEO and the ground station is around 10,000 to 20,000 km, and the distance between the LEO and the earth station is between 160 and 2000 km. The operating frequencies for FSS (uplink) are 14 to 14.5 GHz, FSS downlink is 12.5 to 12.75 GHz, BSS uplink is 17.3 to 17.8 GHz, EESS is 13.4 to 13.75 GHz, and radar is 13.4 to 14 GHz. The gain of the receiver antennas is 35 dBi, and the gain of the transmitter antenna at 12.5 GHz is 25 dBi, at 13.5 GHz is 26.31 dBi, at 14.5 GHz is 26 dBi, and at 17.5 GHz is 22 dBi. To theoretically understand FSPL, the distances between the transmitter and receiver antennas are varied from 5000 to 40,000 km and from 1 to 2000 km, respectively. Figure 17 and Figure 18 display the graphs of the FSPL.

Figure 17a shows the FSPL as the distance between the transmitter and receiver antennas varies from 5000 to 40,000 km, with a receiver gain of 35 dBi. Similarly, Figure 17b,c calculate the FSPL by varying the distance between the transmitter and receiver antennas from 5000 to 40,000 km, with a receiver gain of 40 dBi in Figure 17b and 50 dBi in Figure 17c. It is found that when the receiver gain is 50 dBi, the obtained FSPL is reduced. It is important to note that FSPL increases with distance.

Figure 18a–c show the FSPL attained when the distance between the transmitter and receiver antenna is varied from 1 to 2000 km, while the receiver gain is 35 dBi, 40 dBi, and 50 dBi, respectively. When compared, it is clear that the FSPL decreases as the gain increases, while the FSPL increases as the distance increases. When comparing Figure 17 and Figure 18, it is clear that FSPL is lower in Figure 18, due to the shorter distance between the transmitter and receiver. As a result, FSPL increases with distance, while FSPL decreases with gain and distance.

### 3.4. Link Budget

The link budget is used to calculate the power received at the receiver based on the output power of the transmitter. To calculate the link budget of a system, one must first understand all of the signal’s gains and losses. The signal reliability can be determined for various distances by adjusting the distance between the transmitter and receiver antennas. The link budget can be computed using Equation (5),

(5) $$\;Link\;Margin=Antenna\;power\;\left(A_{p}\right)-Required\;power\;(R_{p})$$

The antenna power is given as:

(6) $$A_{p}\left(dB\right)=P_{Tx}+G_{Tx}+G_{Rx}-L_{F}-P_{L}\;$$

where *P_Tx_* and *G_Tx_* are power and gain of the transmitter, respectively, *G_Rx_* is the receiver’s gain, and *L_F_* and *P_L_* denote free-space loss and polarization mismatch, respectively. The transmitter’s power is 0 dBm, and its gain is 25 dBi at 12.5 GHz, 26.31 dBi at 13.5 GHz, 26 dBi at 14.5 GHz, and 22 dBi at 17.5 GHz. *L_F_* can be calculated using Equation (7),

(7) $$L_{F}=20log\left(\frac{4\pi d}{\lambda }\right)$$

where *d* is the distance and *λ* is the wavelength. The required power, *R_p_*, is expressed as Equation (8),

(8) $$R_{P}\;\left(dB\right)=\frac{E_{b}}{N_{0}}+KT_{0}+B_{r}$$

The value of *E_b_*/*N*_0_ is 9.6 dB as per phase shift keying (PSK), *K* represents the Boltzman constant, and *T*_0_ represents absolute zero temperature. *B_r_* is the bit rate, which is equivalent to 12 Mbps.

**Figure 19:**
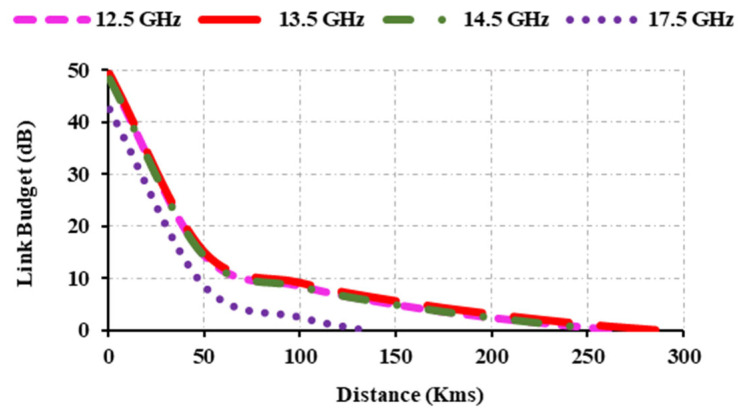
Link budget analysis.

Figure 19 displays the data transmission range. At 12.5 GHz, the obtained data transmission range is up to 269 km, while at 13.5 GHz, it is 290 km. From the figure it can be seen that the attained data transmission range at 14.5 GHz is 260 km. The achieved data transmission range is 135 km at 17.5 GHz. Hence, the data transmission range varies according to the gain and frequency. As the frequency increases, the data transmission range decreases; however, if the gain is high in the upper frequency and very low in the lower frequency, the data transmission range increases.

## 4. Performance Comparison

The performance characteristics of the proposed antenna are compared (see Table 2) to previous research [19,20,21,22,23,24,25,26,27] in the field. The unit cell size, number of elements, aperture size, operating frequency, obtained phase, peak gain, 1 dB gain bandwidth, side lobe level, and cross-polarization of the proposed antenna at 13.5 GHz are compared with the existing works, and the outcomes are listed below:

The proposed antenna has a smaller unit cell than the existing reflectarray antennas [20,21,24,25,27]. It is 30.56% smaller than [20,25,27], 84% more compact than [21], 48.98% more miniature than [24] in terms of mm, and smaller in size than [21,24,27] in terms of wavelength.

The developed reflectarray aperture is smaller than in the previously reported reflectarray works [3,19,21,26]. It is 34.76% more compact than [19], 41.69% smaller than [21], 39.51% more miniature than [3,26] in terms of mm, and smaller in size than [27] in terms of wavelength.

References [3,21] use fewer elements than the proposed antenna, but the aperture size is larger than that of the reflectarray antenna.

The proposed antenna has a wider operating bandwidth than in previous studies [19,20,21,22,24,26,27]. The bandwidth of the developed antenna is 4.75 GHz larger than [19], 4 GHz wider than [21], 2 GHz wider than [22], 3.5 GHz larger than [24], 4 GHz wider than [26], and 4.725 GHz broader than [27].

- The achieved phase response is higher than in previous studies [3,12,19,20,21,22,23,25,26,27]. The phase of the developed antenna is 31.73% larger than [19,22], 22.49% wider than [20], 27.71% larger than [3,12,21,25], 45.78% larger than [23], and 34.74% greater than [26].
- The proposed antenna has a higher peak gain than in previous studies [3,12,19,21,22,23,24,25,26,27]. It is 1.1 dBi greater than [3,25], 7.1 dBi greater than [12], 4.7 dBi greater than [19], 2.6 dBi greater than [21], 2.3 dBi greater than [22], 1.2 dBi, 5.92 dBi greater than [24], and 1.3 dBi greater than [27].
- Side lobe levels are lower than in previous literature [3,12,19,22,25,26], and cross-polarization levels are lower than in references [3,19,20,21,22,23,25,26,27].
- The proposed antenna achieves a 1 dB gain bandwidth of 15.4%, surpassing previous research [21,22,24].
- The obtained aperature efficiency is 44.4%, which is higher than [3,19,21,23,24,26].
- The proposed antenna’s FSPL and link budget are computed to assess signal strength loss and transmission range.

Thus, the proposed antenna is more suitable for remote sensing, environmental mointoring, and satellite applications such as FSS, EESS, and BSS. In the future, metamaterial-based reflectarrays could enable intelligent networking in smart cities by delivering energy-efficient, cost-effective solutions for the Internet of Things (IoT) [28,29,30]. Additionally, multi-antenna and metasurfaces-based reflectarray antennas can support high-throughput satellite systems and allow for efficient beam steering for multiple users on earth or in space [31,32]. They are also useful for secure communications, radar systems, and anti-jamming technologies [33,34].

## 5. Conclusions

A 21 × 21 reflectarray antenna is constructed on the Rogers RT Duroid 5800 substrate for Ku-band applications. The length of the vertical strips is adjusted to achieve the required phase shift in the proposed unit cell. The proposed antenna has a peak gain of 26.31 dBi with an aperture efficiency of 44.4% and maintains a gain greater than 20 dBi from 12 to 18 GHz. The developed antenna is a wideband reflectarray antenna with a fractional bandwidth of 40%. The side lobe levels are less than −15 dB in the E and H planes, with a minimum of −18.5 dB at 13.5 GHz. The 441 elements are arranged in a mirror fashion to produce cross-polarization levels less than −21.46 dB and −25.27 dB at the E and H planes, respectively. The minimum cross-polarization level is −26.03 dB at 13.5 GHz. Furthermore, the constructed reflectarray antenna has a 15.4% 1 dB gain bandwidth. FSPL and link budget calculations are performed theoretically, and it is concluded that as the distance between the transmitter and receiver antennas decreases, the FSPL decreases and the distance covered by the antenna increases. Furthermore, as the gain of the antenna increases, the FSPL decreases while the distance covered by the antenna increases. Thus, the proposed antenna is suitable for applications like remote sensing, environmental monitoring, FSS, BSS, EESS, and maritime satellite communication.

## Figures and Tables

**Table 1:** Structural parameters of the single element.

Notation	Explanation	Parametric Value (mm)
*L*	Length of the single element	10
*W*	Width of the single element	10
*S_V_*	Length of the vertical middle strip	9.5
*S*_1*a*_	Length of the first right strip	6.175
*S*_1*b*_	Length of the first left strip	6.175
*S*_2*a*_	Length of the second right strip	4.75
*S*_2*b*_	Length of the second left strip	4.75
*S_H_*	Length of the horizontal strip	0.5
*C*_1*a*_	Diameter of the circle on first right strip	1
*C*_1*d*_	Diameter of the circle on second left strip	0.6

**Table 2:** Comparison between the proposed work and existing reflectarray works.

Ref.	Unit Cell Dimensions (mm^2^)	Unit Cell Dimensions (*λ*^2^)	Number of Elements	Aperture Size (mm^2^)	Aperture Size (*λ*^2^)	Frequency (GHz)	Phase (Degree)	Peak Gain (dBi)	Side Lobe Level (dB)	Cross-Polarization (dB)	1 dB Gain Bandwidth (%)	Aperture Efficiency (%)
[3]	6 × 6	0.14 × 0.14	228	270 × 270	6.57 × 6.57	7.3–17	360	25.1	–10	–20	-	40
[12]	4.3 × 4.3	0.36 × 0.36	100	-	-	25–33.6	360	19.2	–10	-	30.7	-
[19]	10 × 10	0.31 × 0.31	676	260 × 260	8.02 × 8.02	9.25–10.5	340	21.66	–10	25	-	18
[20]	12 × 12	0.26 × 0.26	221	-	-	6.557, 10	386	26.5	–15	–15	-	-
[21]	25 × 25	0.5 × 0.5	121	275 × 275	5.5 × 5.5	6–8	360	23.7	-	–18	8.1	43.4
[22]	10 × 10	0.27 × 0.27	441	210 × 210	5.6 × 5.6	8–12	340	24	–19	–22	15.3	46
[23]	9 × 9	0.36 × 0.36	315	189 × 189	7.56 × 7.56	12–18	270	25.5	–20	–20	-	40
[24]	14 × 14	0.42 × 0.42	121	154 × 154	4.62 × 4.62	9–11.5	550	20.38	-	-	11.6	33
[25]	12 × 12	0.28 × 0.28	225	180 × 180	4.12 × 4.12	7–14	360	25.2	–15	–15	-	50
[26]	10 × 10	0.3 × 0.3	729	270 × 270	8.1 × 8.1	9–11	325	26.1	–16	–23	18	40.3
[27]	12 × 12	0.519 × 0.519	1517	492 × 444	21.28 × 19.2	12.975–14.25	400	25	-	25	-	-
Prop.	10 × 10	0.4 × 0.4	441	210 × 210	8.4 × 8.4	12–18	498	26.3	–18.5	–26.03	15.4	44.4

## Data Availability

The data presented in this study are available on request from the corresponding author.
